# The influence of KaiA mutations on its function in the KaiABC circadian clock system

**DOI:** 10.1016/j.dib.2018.03.032

**Published:** 2018-03-12

**Authors:** Qiang Chen, Sen Liu, Liting Yang, Lingya Zhang, Jinkui Li

**Affiliations:** a Hubei Key Laboratory of Tumor Microenvironment and Immunotherapy, Medical College, China Three Gorges University, Yichang 443002, China; b National "111" Center for Cellular Regulation and Molecular Pharmaceutics & Institute of Biomedical and Pharmaceutical Sciences, Hubei University of Technology, Wuhan 430068, China

**Keywords:** KaiA, Circadian clock, Protein structure and function, Domain-swapping, Function switching

## Abstract

The core oscillator of the circadian clock of cyanobacteria consists of three proteins, KaiA, KaiB, and KaiC. The KaiABC oscillator can be re-constituted in vitro with the purified proteins in buffer containing ATP and Mg^2+^. The interaction between KaiA and KaiC has not been well studied. In this article, different KaiA mutants were designed and used to elucidate the influence of KaiA structure on its function in the in vitro system. Molecular dynamics simulations were adopted to study the structural flexibility of KaiA homodimer. The data presented in this article provide further experimental supports to our work in Chen et al. (2017) [1].

**Specifications Table**

**Table:** 

Subject area	*Biology, Chemistry*
More specific subject area	*Protein structure and function*
Type of data	*Image, graph, figure*
How data was acquired	*The elution profiles of the proteins were collected using a ÄKTA*
	*Purifier 100 (GE) system. The images of the SDS-PAGE gels were taken on a gel imaging system (Kodak, Gel Logic 200).The molecular dynamics simulations were performed in NAMD on a GPU server.*
Data format	*Raw, analyzed*
Experimental factors	*The clock proteins were expressed in E. coli and purified. Then the proteins were mixed under different combinations and incubated at 30 °C to collect samples at indicated time points. The samples were analyzed with 8% SDS-PAGE gels to analyze the phosphorylation of KaiC.*
Experimental features	*Test KaiA's function using SDS-PAGE and molecular dynamics simulations*
Data source location	*College of Medical Science, China Three Gorges University, Yichang, China*
Data accessibility	*Data are presented in the article*

**Value of the data**

• This data article presents versatile protein design strategies to study the structure-function relationship of KaiA.
• The data article focused on the analysis of the in vitro KaiABC system using SDS-PAGE.
• This dataset could be a useful reference to study the relationship between protein structural flexibility and functional dynamics.

## 1 Data

Circadian rhythms are the ~ 24 h cycles of the physiological processes in living beings on Earth. The cyanobacterial circadian clock consists three proteins, namely KaiA, KaiB, and KaiC. KaiA binds to KaiC through the interaction between KaiA's C-terminal domains and KaiC's C-terminal tail [2]. However, the details of KaiA exerts its function are still not known very well, especially how KaiA does functional switch between active and in-active. Very recently, Tseng et al. presented a possible mechanism that KaiA gets auto-inhibited [3]. Our work in this article and in Ref. [1] provided further evidence and clues to the function regulation of KaiA.

**Fig. 1:**
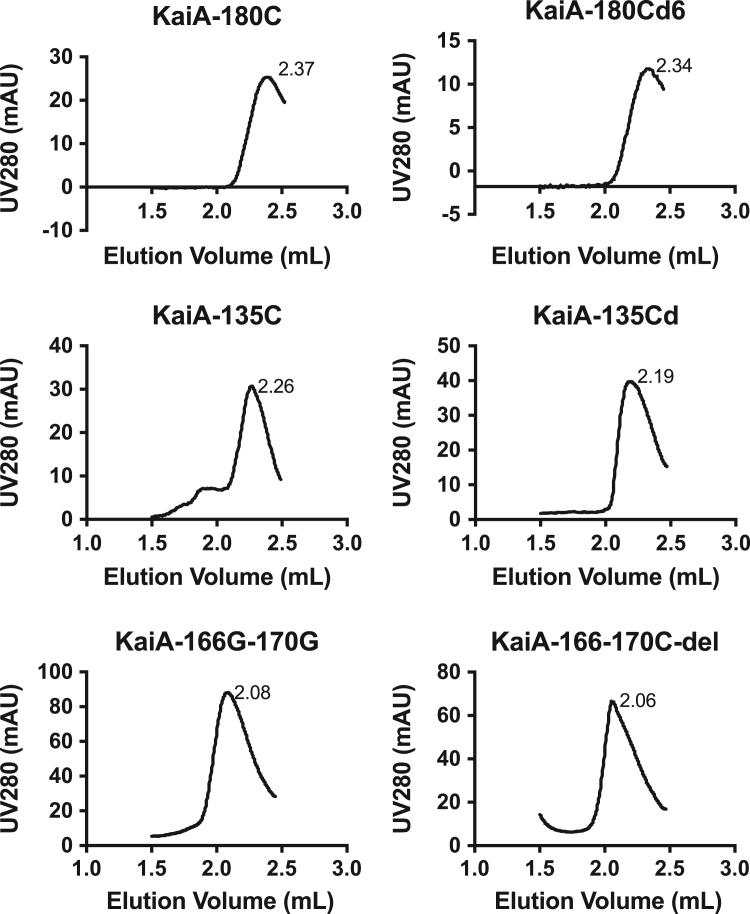
The elution profiles of KaiA constructs from the size exclusion chromatography. The void volume was determined to be 1.4 mL with blue dextran, and the column volume was 2.4 mL, which are set as the cutoffs of the curves. KaiA-180C: the C-terminal domain of KaiA; KaiA-180Cd6: the concatenated form of KaiA-180C; KaiA-135C: the central domain and the C-terminal domain; KaiA-135Cd: the concatenated form of KaiA-135C; KaiA-166G-170G: KaiA with the mutations Y166G and Y170G; KaiA-166-170-del: KaiA with the deleted segment from the residue 166 to the residue 170.

**Fig. 2:**
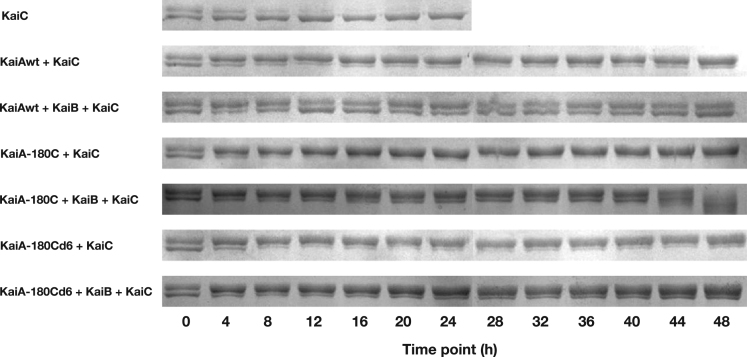
The SDS-PAGE analysis of the phosphorylation states of KaiC in the reconstitution system for KaiAwt, KaiA-180C and KaiA-180Cd6. In each lane, the upper band was treated as the phosphorylated KaiC and the lower band as the de-phosphorylated KaiC. Heavily smeared lanes were excluded from the quantitative analysis.

**Fig. 3:**
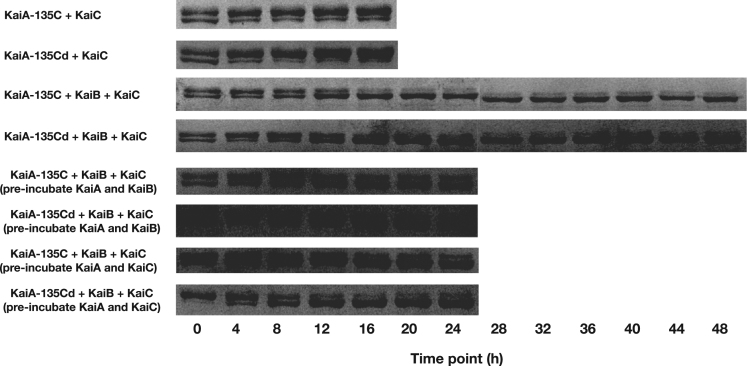
The SDS-PAGE analysis of the phosphorylation state of KaiC in the reconstitution system for KaiA-135C and KaiA-135Cd. In each lane, the upper band was treated as the phosphorylated KaiC and the lower band as the de-phosphorylated KaiC. In the pre-incubation tests, KaiA and KaiB (or KaiC) were incubated for 12 h at 30 °C before KaiC (or KaiB) was added.

**Fig. 4:**
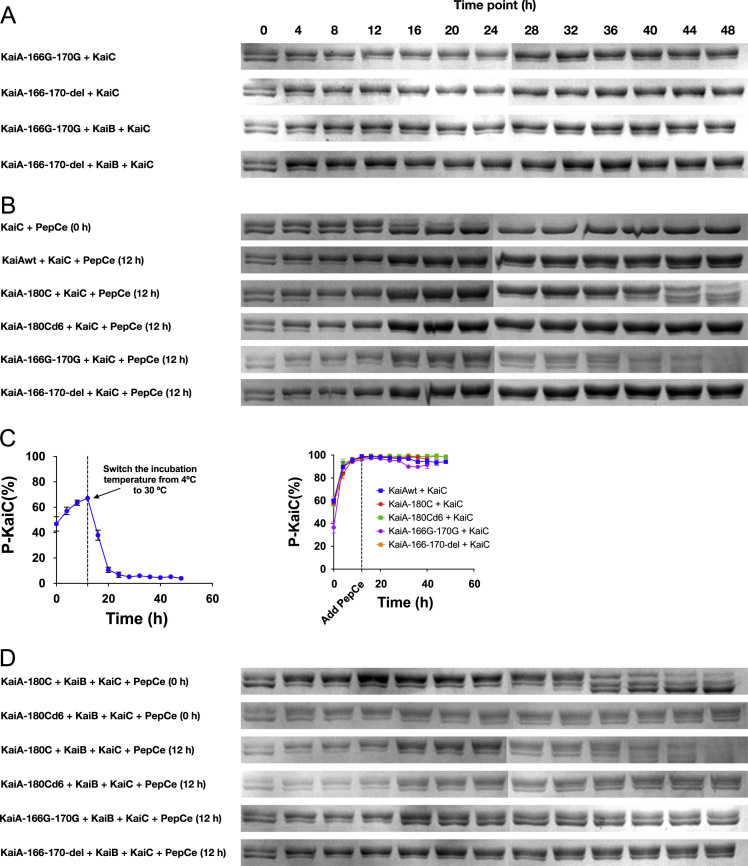
The SDS-PAGE analysis of the phosphorylation state of KaiC in the reconstitution system. In each lane, the upper band was treated as the phosphorylated KaiC and the lower band as the de-phosphorylated KaiC. The lanes with smeared bands were excluded. The time points adding PepCe to the mixtures are in the parentheses. The molar amount of PepCe was equal to the molar amount of the single KaiA C-terminal domain in the corresponding system. (**A**) The gels for KaiA-166-170-del and KaiA-166G-170G. (**B**) The gels for PepCe adding into KaiC or KaiA plus KaiC proteins. (**C**) The quantitative analyses of the gels in (**B**). (**D**) The gels for PepCe adding to KaiA plus KaiC proteins. The error bars in (C) represent the standard error (SD) from two tests.

**Fig. 5:**
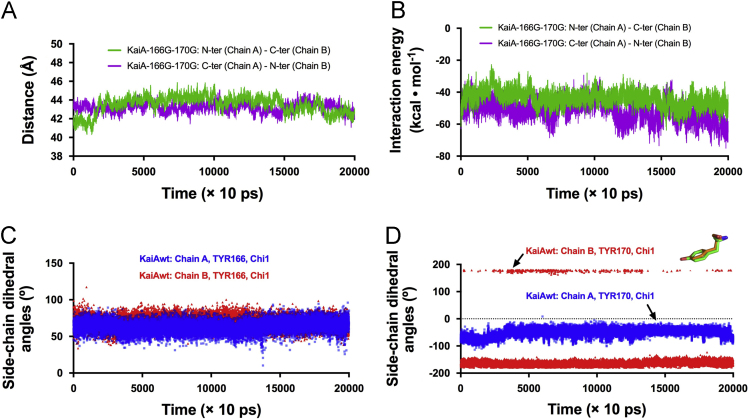
The molecular dynamics simulation of KaiA-166G-170G and KaiAwt. (**A**) The distances of the mass centers of the contacted domains in KaiA-166G-170G. N-ter: The N-terminal domain; C-ter: The C-terminal domain. (**B**) The domain-domain interaction energies of the contacted domains in KaiA-166G-170G. (**C**) The side-chain dihedral angles of the two TYR166 residues in the chain A and B in KaiAwt. The Chi1 angle of tyrosine is formed by the atoms N-CA-CB-CG. (**D**) The side-chain dihedral angles of the TYR170 residues in the chain A and B in KaiAwt. The TYR170 residue in the chain B had positive Chi1 angles more often between 300 and 140 ns. The cartoon represents two TYR170 residues in the chain B from two models with different Chi1 angles. Green: Chi1 = − 166.9°; Orange: Chi1 = 179.2°.

Using the Kai proteins of the cyanobacterium *Synechococcus elongatus* PCC 7942 (*S. e.* PCC 7942), we set up the in vitro system to study the oscillation of KaiC's phosphorylation status based on SDS-PAGE. To study the structure-function relationship of KaiA, we designed different KaiA constructs. In this article, we presented our experimental data to further support our work in [1]. In Fig. 1, we presented the elution profiles of different KaiA constructs. In Fig. 2, we showed the SDS-PAGE gel images for testing the function of the wild-type KaiA (KaiAwt), the C-terminal domain of KaiA (KaiA-180C), and its concatenated form (KaiA-180Cd6). Fig. 3 showed the SDS-PAGE gel images for testing the function of the KaiA constructs containing the central domain and the C-terminal domain (KaiA-135C) and a concatenated form of KaiA-135C (KaiA-135Cd). Fig. 4 contained the SDS-PAGE gel images and analytic graphs of the KaiA constructs with modified central domains (KaiA-166–170-del and KaiA-166G-170G) and the independent central domain (PepSe). Fig. 5 presented the 200 ns molecular dynamics simulation of KaiA-166G-170G and KaiAwt.

## 2 Experimental design, materials and methods

### 2.1 Protein expression and purification

The expression and purification of KaiA, KaiB, and KaiC were similar with our previous report [2]. Briefly, all proteins were expressed as GST-tagged proteins, and then the GST tags were removed with PreScission Protease. The tag removed proteins were further purified with Hitrap FF Q columns. All protein coding sequences were verified with DNA sequencing.

### 2.2 Size exclusion chromatography

A Superdex 200 Increase 3.2/300 column (GE Healthcare) was used for evaluating the oligomerization states of the proteins. The proteins were prepared in the reconstitution buffer (50 mM Tris–HCl pH 8.0, 150 mM NaCl, 5 mM ATP, 5 mM MgCl_2_, and 0.01% Tween-20) and loaded to the column for analysis separately at room temperature with a flow rate of 0.01 mL/min.

### 2.3 *in vitro* reconstitution assay

The reconstitution assay was similar with our previous report [2]. Briefly, the purified proteins were incubated in the reaction buffer (50 mM Tris–HCl pH 8.0, 150 mM NaCl, 5 mM ATP, 5 mM MgCl_2_, and 0.01% Tween-20) at the ratio of KaiA:KaiB:KaiC = 1:1:2 (m/v). The reaction system was incubated at 30 °C and samples were collected at indicated time points. Finally, the samples were analyzed with 8% SDS-PAGE gels.

### 2.4 Evaluation of the SDS-PAGE gels

The SDS-PAGE gels were analyzed using Image J [4] with the gel analysis protocol as described in our published protocol as described in our published work [5].

### 2.5 Molecular dynamics simulation

The molecular dynamics simulation was performed in NAMD [6] as previous [2] using the KaiA homo-dimer structure in 5C5E (PDB ID). Periodic water boxes were added to wrap the protein with 10 Å of boundary distances. Na^2+^ and Cl^−^ were added to 0.15 mol/L and counteracted the net chargers of the system. The Charmm parameters from c35b2_c36a2 were used, and the smooth particle-mesh Ewald (PME) method was enabled. The data were analyzed in VMD [7].
