# Supplementary material for: The influence of KaiA mutations on its function in the KaiABC circadian clock system
Source: Data Brief. 2018 Mar 12;18:241–7. doi: 10.1016/j.dib.2018.03.032 (PMC5996256; doi:10.1016/j.dib.2018.03.032)
Supplement: Supplementary file 1 — Transparency document [file mmc1.docx]

Conflict of interest

Manuscript No.: DIB-D-17-01108
Title: The influence of KaiA mutations on its function in the KaiABC circadian clock system
Journal Title: Data in Brief
Corresponding Author: Dr. Sen Liu

Authors’ statement: There is no conflict of interest.
